# Knowledge and Information Exposure About Family Planning Among Women of Reproductive Age in Informal Settlements of Kira Municipality, Wakiso District, Uganda

**DOI:** 10.3389/fgwh.2021.650538

**Published:** 2021-05-25

**Authors:** Catherine Birabwa, Dennis Chemonges, Moses Tetui, Mazen Baroudi, Fredinah Namatovu, Joseph Akuze, Fredrick Makumbi, Tonny Ssekamatte, Lynn Atuyambe, Alison Hernandez, Maquins Odhiambo Sewe

**Affiliations:** ^1^Department of Health Policy, Planning and Management, Makerere University School of Public Health, New Mulago Hospital Complex, Kampala, Uganda; ^2^Department of Programs, Population Services International Uganda, Kampala, Uganda; ^3^Department of Epidemiology and Biostatistics, Makerere University School of Public Health, New Mulago Hospital Complex, Kampala, Uganda; ^4^School of Pharmacy, Waterloo University, Waterloo, ON, Canada; ^5^Department of Epidemiology and Global Health, Umeå University, Umeå, Sweden; ^6^Faculty of Epidemiology and Population Health, London School of Hygiene and Tropical Medicine, London, United Kingdom; ^7^Department of Disease Control and Environmental Health, Makerere University School of Public Health, New Mulago Hospital Complex, Kampala, Uganda; ^8^Department of Community Health and Behavioural Sciences, Makerere University School of Public Health, New Mulago Hospital Complex, Kampala, Uganda; ^9^Department of Public Health and Clinical Medicine, Sustainable Health Section, Umeå University, Umeå, Sweden

**Keywords:** knowledge, media exposure, family planning, informal settlements, Uganda

## Abstract

**Introduction:** A high unmet need for family planning (FP) prevails in sub-Saharan Africa. Knowledge, awareness creation, and ensuring accessibility are frequently used to increase FP uptake. However, evidence on knowledge or information dissemination about FP among marginalized populations in urban settings in Africa is limited. This study explored the knowledge of FP methods, media exposure, and contact with FP providers among women from an informal settlement in Uganda.

**Methods:** Using a cross-sectional study design, we interviewed 626 women aged 15–49 years living in informal settlements of Kira municipality, selected through multistage sampling. Using a standard questionnaire, data was collected on socioeconomic characteristics, knowledge of FP methods, and access to media FP messages among others. Binomial log-linear regression was used to assess disparities in exposure to media FP messages or provider information. Data were analyzed using STATA version 14, at a 5% level of statistical significance.

**Results:** Nearly all women in the survey were aware of FP methods (99.7%). On average, each woman was aware of 10 FP methods. The most commonly known methods were male condoms (98.2%), injectables (97.4%), and the oral contraceptive pill (95.2%). Use of any contraceptive was found among 42.7% of respondents. Exposure to media was found in 70.6% of the respondents, mostly through television (58.5%) and radio (58.3%). Discussing FP with a provider was significantly associated with media exposure (aPR 1.4, 95% CI: 1.24–1.56). Less than 50% of women who were not using FP had contact with an FP provider. Women in union (aPR 1.6, 95% CI: 1.01–2.68) and those with access to media messages (aPR 2.5, 95% CI: 1.37–4.54) were more likely to have contact with a provider to discuss FP.

**Conclusion:** There is high general awareness about FP methods and media exposure, but method use was low. Further exploration of women's understanding of FP methods and the fit between existing education programs and FP knowledge needs in this urban setting should be conducted. The potential for mobile health solutions in this urban population should be explored. Future studies should focus on the knowledge and understanding of FP among unmarried and nulliparous women and those with no access to media information.

## Introduction

The world is fast urbanizing, and it is estimated that, by 2050, two thirds of the global population will be urban (1, 2). Sub-Saharan Africa is the fastest urbanizing region in the world, yet about 70% of its urban population resides in informal settlements (3, 4). Urban areas have conventionally been reported to have higher modern contraception use and lower unmet need for family planning (FP), attributable to better exposure to, proximity to, and diversity of services (5, 6). However, previously reported rural–urban differences are decreasing, and growing intra-urban inequalities are driving poor outcomes among urban residents (7). The poor in informal urban settlements suffer the greatest brunt of health inequity occasioned by a poor living environment, social exclusion, and fewer opportunities for socioeconomic empowerment (8, 9). Unfortunately, most health indicators, including FP measures, of this subpopulation are often masked in general urban averages (5).

In Uganda, the urban population has grown over the years and is currently estimated at 9.4 million (10). This, in part, is attributed to high fertility coupled with an unmet need for FP of about 23% and rural–urban migration (6). Furthermore, poor contraceptive behaviors, such as FP discontinuation, and missed opportunities for counseling and promoting FP uptake have also been reported to limit effectiveness of FP (11). Fertility among residents of informal settlements is high with poorer birth outcomes compared with well-off urban dwellers or rural counterparts (3, 12–14). Studies also indicate that living in informal settlements negatively affects women's ability to control their fertility (15–17). Therefore, access to and utilization of FP information and services is important to persons living in informal settlements. This is critical in the quest for universal access to sexual and reproductive health information, education, and services for the 2030 Agenda (18).

Knowledge of FP is nearly universal in Uganda at more than 98% in urban or rural areas, and health providers are the most trusted source of FP information (6, 19). Some studies show positive correlation between FP knowledge and sociodemographic factors, such as marital status and gender (20), although others indicate no such association (6). Having knowledge of FP is expected to modify contraceptive behaviors, and earlier studies report a positive relationship between knowledge and media exposure to FP messages with increased acceptance and use of contraceptives (21–24). Media exposure to FP information among Ugandans is mainly through radio, television, and newspaper at 65, 20, and 11%, respectively (6). Residing in urban areas, age, and being educated or employed are associated with higher media exposure (6, 25, 26). However, few studies have explored knowledge of and information dissemination for FP in residents of informal settlements in Uganda. Lack of such context-specific information limits the capacity of programming and policy making to effectively meet the unmet need for FP in these vulnerable subpopulations.

The aim of this study was to assess the knowledge of FP methods and the level and determinants of FP information dissemination through mass media among all women and FP providers among non-users of contraception in an informal urban settlement in Wakiso, Uganda. This information informs efforts geared toward creating demand and strengthening women's ability to independently make and act on decisions regarding contraceptive use (contraceptive autonomy) through information.

## Materials and Methods

### Study Design and Setting

We used a cross-sectional study design among informal settlements in Kira municipality, Wakiso district, Uganda. Kira municipality is located ~5.3 km by road, East of Kampala, and is made up of three divisions, namely; Kira, Bweyogerere, and Namugongo, which occupy a total land area of about 98.83 km^2^. The municipality is characterized by a high population, which has, in turn, compromised physical planning and effective delivery of social services, including health care. The rapid population growth in Kira municipality has resulted in the growth of informal settlements, whose inhabitants have low socioeconomic status and reside in poor household structures.

### Study Population and Data Collection

The survey targeted sexually active women of reproductive age (15–49 years) who were living in the informal settlements of Kira. Women who were not residents of Kira municipality and those who had not stayed in Kira for at least 6 months prior to the survey were not included. A sample size of 627 was computed using the Kish Leslie formula for cross-sectional studies. A prevalence of 52.1% for modern contraceptive use in an urban setting was used (6) at a 95% level of confidence and 5% margin of error. This yielded a minimum sample size of 377. This was adjusted for 10% non-response, bringing the sample size to 418. In addition, a design effect of 1.5 was applied to cater to the distribution of the sample across two divisions that had informal settlements in Kira municipality, giving a final sample size of 627 participants. However, data was collected from 626 respondents on whom analysis was performed.

Participants were selected through multistage sampling. Two divisions were purposely selected based on the presence of informal settlements. Within these two divisions, eight villages were found within the informal settlements of which four were randomly selected to be study sites. A list of enumeration areas (EAs) used by the Uganda Bureau of Statistics was then used to identify EAs that fell within the four selected villages. A total of 65 EAs were obtained of which 13 were randomly selected. Within these EAs, a listing of all households with eligible women was obtained. A simple random sample of households, equal to the required sample size, was selected from the listed households. The sample size was equally distributed across the 13 EAs, giving an average of about 49 respondents from each.

A standard questionnaire by Performance Monitoring and Accountability (PMA) was used. This PMA questionnaire is consistent with the demographic health survey women's questionnaire, which has already been validated. Data on sociodemographic characteristics of women, birth history, pregnancy, sexual and contraception history, and women's contraceptive decision-making power was collected. Contraception history captured data on knowledge of FP methods, ever and current use of contraception, knowledge of source and reported exposure to FP information through media channels, and community/facility counseling services, among other information. The tool was uploaded on the KoboCollect mobile application, which was used by the data collectors. The research assistants uploaded the collected data to the server daily. Upon submission of the data to the server, the investigators and the data managers conducted quality control checks on key variables, such as age to ascertain their correctness.

### Quality Control

Research assistants were trained on the research protocol and ethical issues surrounding the study to ensure quality data collection. A pretest of the data collection tools was conducted in Katanga, an informal settlement located in Uganda's main city of Kampala. Katanga was selected as the ideal pretest site because it has similar characteristics as the selected EAs in Kira municipality, such as being densely populated with a majority of inhabitants living below the poverty line in poor housing structures.

### Measures

Knowledge of FP was defined as ever hearing about at least one (any) FP method. Women were asked if they had ever heard about 13 methods of FP, coded as one (yes) and zero (no). Exposure to FP information was assessed for the general population of women surveyed and also specific for women who were not using FP. Among all women surveyed, media exposure was defined as the percentage of all women who had heard or seen an FP message on the radio, television, in a newspaper or magazine, or on a mobile phone in the past few months prior to the interview or in none of the four media sources. A dichotomous variable was generated and coded as being exposed (one) or not exposed (zero). For the women who were not using FP, contact with FP providers was used to assess reach of FP information. This was defined as the percentage of women who were not using contraception, who were visited by a field worker who discussed FP, who visited a health facility and discussed FP, who visited a health facility but did not discuss FP and who did not discuss FP with a field worker during the 12 months preceding the survey.

We also included sociodemographic and other factors to examine variations in access to FP information through media. These were woman's age, education, marital status and decision-making power, ever giving birth, parity, fertility preferences, and wantedness of the last birth the woman had as well as contact with an FP provider. The woman's decision-making power was measured by her ability to (1) tell her partner about contraception use and (2) tell her partner if she did not desire to have sexual intercourse as well as her ability (3) to use contraception when desired and (4) to avoid sexual intercourse if she did not desire to have it. Women were categorized as empowered if they were able to do all four actions. Contact with FP providers included being visited by a field FP worker and discussing FP with a health worker when the woman visited a health facility.

Contraceptive use and discontinuation as well as intention to use FP were explored in relation to media exposure. Contraceptive use was measured as women who reported use of FP at the time of the survey (currently using FP). Discontinuation referred to women who had stopped using their current method of FP at the time of the survey, and intention to use FP was defined as women who reported not using FP at the time of the survey but were planning to use FP in the future.

### Data Analysis

Descriptive statistics, including frequencies and proportions, were used to summarize the categorical variables and examine their distribution by level of media exposure to FP messages. Media exposure was assessed by specific channel and as a composite of all channels. The composite variable was used to assess disparities in access to FP information through media and to explore the relationship between exposure to FP information through media and contraceptive use, demand for FP, contraceptive intentions, and discontinuation. Bivariate and multivariate binomial log-linear regression analysis (27) was performed using prevalence ratios to examine the relationship between media exposure and selected variables at a 5% level of significance. The multivariable model included variables that were significant in the bivariate analysis. Variation in contact of women who were not using contraception with FP providers was assessed using binomial log-linear regression. All analyses were conducted using STATA 14.

## Results

### Background Characteristics of Respondents

A total of 626 women were interviewed in the survey. The mean age of the participants was 28.1 (±7.6) years. The youth, aged 15–24 years, represented 36.1% of all women in the survey (Table 1). Most (55.9%) respondents had attended secondary or a higher level of education, and nearly 75% were currently married or living with a man.

**Table 1:** Characteristics of the study population.

**Variable**	**Category**	**Number (%)**
Currently using FP	No	359 (57.3)
	Yes	267 (42.7)
Intention to use	No	25 (18.5)
	Yes	110 (81.5)
Discontinued FP use	No	229 (56.8)
	Yes	174 (43.2)
Age (years)	15-24	226 (36.1)
	25–34	270 (43.1)
	35–49	130 (20.8)
Education level	No education	39 (6.3)
	Primary	235 (37.8)
	Secondary and higher	347 (55.9)
Marital status	Formerly/never in union	158 (25.4)
	Currently married/in union	463 (74.6)
Ever given birth	No	117 (18.7)
	Yes	509 (81.3)
Parity	Only 1	138 (27.1)
	2–3	235 (46.2)
	>3	136 (26.7)
Wantedness of last birth	No child	66 (12.97)
	Later	96 (18.86)
	Then	344 (67.58)
Fertility preference	No child/can't get pregnant	142 (24.5)
	Another child/undecided	424 (73.1)
Contact with FP	No	293 (46.8)
provider	Yes	333 (53.2)
Woman's decision-making power	No power	61 (9.7)
	Have power	565 (90.3)

### Knowledge of FP Methods and Source

Nearly all (99.7%) women in the survey reported ever hearing about at least one (any) FP or modern contraceptive method (Table 2). After removing use of condoms (given their dual role of FP and prevention of HIV/STIs), awareness of FP methods remained high at 99.5%. On average, each woman had ever heard of 10 methods of FP. The most commonly known methods were male condoms (98.2%), injectables (97.4%), oral contraceptive pills (95.2%), and implants (90.1%), and the least known were spermicides (14.5%) and the contraceptive diaphragm (22.2%). More than 90% of all women reported to know where they could obtain modern FP methods.

**Table 2:** Knowledge of contraceptive methods and where to access contraceptive methods.

**Method**	**Number (percent)**
**Any FP method**	**624 (99.7)**
Any FP method besides condoms	623 (99.5)
Any modern method	624 (99.7)
**Long-acting reversible contraceptives**	**574 (91.7)**
Intra-uterine device	508 (81.2)
Implant	564 (90.1)
**Short-acting reversible contraceptives**	**623 (99.5)**
Injectables	610 (97.4)
Oral contraceptive pill	596 (95.2)
Male condom	615 (98.2)
Female condom	446 (71.3)
Spermicides (foam/jelly)	91 (14.5)
**Permanent methods**
Female sterilization	446 (71.3)
Male sterilization	388 (61.98)
**Other modern methods**
Lactational amenorrhea	407 (65.0)
Diaphragm	139 (22.2)
Emergency contraception	472 (75.4)
Standard days/beads	357 (57.0)
**Traditional methods**
Rhythm	394 (62.9)
Withdrawal	545 (87.1)
Mean number of methods known per woman	10.7
**Know where to get FP services**
No	50 (7.99)
Yes	576 (92.0)
Number of women interviewed	626

*The bold values are overall percentages for knowledge of any method, long and short acting methods*.

### Exposure to FP Information

Seventy percent of all women in the survey had been exposed to FP messages, mainly through radio (58.3%) and television (58.5%) (Table 3). The media channel with the lowest exposure was mobile phones at 10%. Women who were using FP at the time of the survey had mostly (67.0%) been exposed through television, and those who had intentions of using FP had been exposed through radio (61.8%). Adolescents and young women (15–24 years) had mostly (53.1%) been reached through television, and women who had contact with FP providers had largely (71.5%) been exposed through radio.

**Table 3:** Exposure to FP information by channel and respondent characteristics.

**Covariate**	**Radio**			**Television**			**Newspaper/magazine**			**Mobile phone**			Overall^*^	
	**No**	**Yes**	**NA**	**No**	**Yes**	**NA**	**No**	**Yes**	**NA**	**No**	**Yes**	**NA**	**No**	**Yes**
**Currently using FP**														
No	53 (38.7)	81 (59.1)	3 (2.2)	47 (34.3)	70 (51.1)	20 (14.6)	99 (72.3)	26 (19.0)	12 (8.8)	121 (88.3)	12 (8.8)	4 (2.9)	39 (28.5)	98 (71.5)
Yes	104 (39.0)	154 (57.7)	9 (3.4)	68 (25.5)	179 (67.0)	20 (7.5)	188 (70.4)	45 (16.9)	34 (12.7)	248 (92.9)	7 (2.6)	12 (4.5)	71 (26.6)	196 (73.4)
**Intention to use FP**														
No	12 (48.0)	11 (44.0)	2 (8.0)	10 (40.0)	12 (48.0)	3 (12.0)	17 (68.0)	4 (16.0)	4 (16.0)	22 (88.0)	2 (8.0)	1 (4.0)	10 (40.0)	15 (60.0)
Yes	41 (37.3)	68 (61.8)	1 (0.9)	37 (33.6)	56 (50.9)	17 (15.5)	82 (74.5)	20 (18.2)	8 (7.3)	99 (90.0)	8 (7.3)	3 (2.7)	29 (26.4)	81 (73.6)
**Discontinued FP use**														
No	89 (38.9)	137 (59.8)	3 (1.3)	59 (25.8)	157 (68.6)	13 (5.7)	160 (69.9)	42 (18.3)	27 (11.8)	208 (90.8)	12 (5.2)	9 (3.9)	55 (24.0)	174 (76.0)
Yes	68 (39.1)	97 (55.7)	9 (5.2)	56 (32.2)	91 (52.3)	27 (15.5)	126 (72.4)	29 (16.7)	19 (10.9)	160 (92.0)	7 (4.0)	7 (4.0)	55 (31.6)	119 (68.4)
**Age (years)**														
15–24years	109 (48.2)	111 (49.1)	6 (2.7)	82 (36.3)	120 (53.1)	24 (10.6)	165 (73.0)	32 (14.2)	29 (12.8)	194 (85.8)	20 (8.8)	12 (5.3)	82 (36.3)	144 (63.7)
25–34years	94 (34.8)	168 (62.2)	8 (3.0)	78 (28.9)	165 (61.1)	27 (10.0)	194 (71.9)	52 (19.3)	24 (8.9)	242 (89.6)	19 (7.0)	9 (3.3)	68 (25.2)	202 (74.8)
35–49years	39 (30.0)	86 (66.2)	5 (3.8)	36 (27.7)	81 (62.3)	13 (10.0)	70 (53.8)	39 (30.0)	21 (16.2)	102 (78.5)	23 (17.7)	5 (3.8)	34 (26.2)	96 (73.8)
**Education level**														
No education	15 (38.5)	21 (53.8)	3 (7.7)	12 (30.8)	22 (56.4)	5 (12.8)	27 (69.2)	7 (17.9)	5 (12.8)	3 2(82.1)	5 (12.8)	2 (5.1)	14 (35.9)	25 (64.1)
Primary	97 (41.3)	130 (55.3)	8 (3.4)	69 (29.4)	138 (58.7)	28 (11.9)	151 (64.3)	38 (16.2)	46 (19.6)	208 (88.5)	13 (5.5)	14 (6.0)	71 (30.2)	164 (69.8)
Secondary and higher	128 (36.9)	211 (60.8)	8 (2.3)	112 (32.3)	204 (58.8)	31 (8.9)	247 (71.2)	77 (22.2)	23 (6.6)	294 (84.7)	43 (12.4)	10 (2.9)	97 (28.0)	250 (72.0)
**Marital status**														
Formerly/never in union	68 (43.0)	83 (52.5)	7 (4.4)	61 (38.6)	80 (50.6)	17 (10.8)	117 (74.1)	21 (13.3)	20 (12.7)	136 (86.1)	14 (8.9)	8 (5.1)	61 (38.6)	97 (61.4)
Currently married/in union	171 (36.9)	280 (60.5)	12 (2.6)	132 (28.5)	284 (61.3)	47 (10.2)	309 (66.7)	100 (21.6)	54 (11.7)	399 (86.2)	46 (9.9)	18 (3.9)	120 (25.9)	343 (74.1)
**Ever given birth**														
No	49 (41.9)	62 (53.0)	6 (5.1)	43 (36.8)	60 (51.3)	14 (12.0)	77 (65.8)	22 (18.8)	18 (15.4)	94 (80.3)	17 (14.5)	6 (5.1)	45 (38.5)	72 (61.5)
Yes	193 (37.9)	303 (59.5)	13 (2.6)	153 (30.1)	306 (60.1)	50 (9.8)	352 (69.2)	101 (19.8)	56 (11.0)	444 (87.2)	45 (8.8)	20 (3.9)	139 (27.3)	370 (72.7)
**Parity**														
Only 1	56 (40.6)	81 (58.7)	1 (0.7)	43 (31.2)	86 (62.3)	9 (6.5)	107 (77.5)	22 (15.9)	9 (6.5)	119 (86.2)	14 (10.1)	5 (3.6)	34 (24.6)	104 (75.4)
2–3	91 (38.7)	141 (60.0)	3 (1.3)	70 (29.8)	146 (62.1)	19 (8.1)	153 (65.1)	60 (25.5)	22 (9.4)	201 (85.5)	27 (11.5)	7 (3.0)	62 (26.4)	173 (73.6)
>3	46 (33.8)	81 (59.6)	9 (6.6)	40(29.4)	74 (54.4)	2 2(16.2)	92 (67.6)	19 (14.0)	25 (18.4)	124 (91.2)	4 (2.9)	8 (5.9)	43 (31.6)	93 (68.4)
**Wantedness of last birth**														
No child	21 (31.8)	41 (62.1)	4 (6.1)	21 (31.8)	36 (54.5)	9 (13.6)	35 (53.0)	22 (33.3)	9 (13.6)	47 (71.2)	18 (27.3)	1 (1.5)	23 (34.8)	43 (65.2)
Later	37 (38.5)	58 (60.4)	1 (1.0)	30 (31.2)	55 (57.3)	11 (11.5)	73 (76.0)	17 (17.7)	6 (6.2)	79 (82.3)	14 (14.6)	3 (3.1)	27 (28.1)	69 (71.9)
Then	134 (39.0)	202 (58.7)	8 (2.3)	100 (29.1)	214 (62.2)	30 (8.7)	243 (70.6)	60 (17.4)	41 (11.9)	316 (91.9)	12 (3.5)	16 (4.7)	89 (25.9)	255 (74.1)
**Fertility preference**														
No child/can't get pregnant	58 (40.8)	77 (54.2)	7 (4.9)	41 (28.9)	84 (59.2)	17 (12.0)	92 (64.8)	24 (16.9)	26 (18.3)	130 (91.5)	5 (3.5)	7 (4.9)	48 (33.8)	94 (66.2)
Another child/undecided	159 (37.5)	256 (60.4)	9 (2.1)	130 (30.7)	254 (59.9)	40 (9.4)	294 (69.3)	85 (20.0)	45 (10.6)	359 (84.7)	49 (11.6)	16 (3.8)	114 (26.9)	310 (73.1)
**Contact with FP provider**														
No	150 (51.2)	127 (43.3)	16 (5.5)	113 (38.6)	136 (46.4)	44 (15.0)	209 (71.3)	27 (9.2)	57 (19.5)	270 (92.2)	3 (1.0)	20 (6.8)	124 (42.3)	169 (57.7)
Yes	92 (27.6)	238 (71.5)	3 (0.9)	83 (24.9)	230 (69.1)	20 (6.0)	220 (66.1)	96 (28.8)	17 (5.1)	268 (80.5)	59 (17.7)	6 (1.8)	60 (18.0)	273 (82.0)
**Woman's decision-making power**														
No power	27 (44.3)	30 (49.2)	4 (6.6)	24 (39.3)	28 (45.9)	9 (14.8)	42 (68.9)	11 (18.0)	8 (13.1)	47 (77.0)	10 (16.4)	4 (6.6)	27 (44.3)	34 (55.7)
Have power	215 (38.1)	335 (59.3)	15 (2.7)	172 (30.4)	338 (59.8)	55 (9.7)	387 (68.5)	112 (19.8)	66 (11.7)	491 (86.9)	52 (9.2)	22 (3.9)	157 (27.8)	408 (72.2)

* *Percentage of all women who heard, saw, or read an FP message on the radio, television, or in a newspaper/magazine, or mobile phone*.

### Contact of Non-users With FP Providers

The study included 222 women who were not using FP at the time of the survey or ever. Of these, 109 (49.1%) were aged between 15 and 24 years, 107 (48.2%) were sexually active, 120 (54.1%) had attained a secondary or higher education level, and 130 (58.56%) were married or in a union at the time of the survey. Table 4 shows that only 26% of the women who were not using FP were reached by a field worker in regard to FP, and 84% of the 115 women who were not using contraception and had visited a health facility reported discussing FP with a health worker. More than 50% of women not using FP were neither reached by a field worker nor told about FP when they visited a health facility.

**Table 4:** Contact of non-users of contraception with FPg providers by respondent characteristics.

**Background characteristics**	**Visited by a field worker**		**Visited HF and discussed FP**		**Overall contact**	
	**Yes *n* (%)**	**No *n* (%)**	**Yes *n* (%)**	**No *n* (%)**	**Yes *n* (%)**	**No *n* (%)**
**Overall**	57 (25.7)	161 (72.5)	96 (83.5)	19 (16.5)	107 (48.2)	115 (51.8)
**Age (years)**					
15–24	21 (19.3)	85 (77.98)	45 (83.3)	9 (16.7)	51 (46.8)	58 (53.2)
25–34	14 (22.95)	47 (77.1)	28 (87.5)	4 (12.5)	29 (47.5)	32 (52.5)
35–49	22 (42.3)	29 (55.8)	23 (79.3)	6 (20.7)	27 (51.9)	25 (48.1)
**Education level**					
No education	6 (35.3)	11 (64.7)	6 (85.7)	1 (14.3)	7 (41.2)	10 (58.8)
Primary	21 (26.3)	58 (72.5)	29 (80.6)	7 (19.4)	34 (42.5)	46 (57.5)
Secondary and higher	28 (23.3)	91 (75.8)	59 (84.3)	11 (15.7)	64 (53.3)	56 (46.7)
**Marital status**					
Formerly/never in union	10 (11.4)	76 (86.4)	27 (72.97)	10 (27.0)	28 (31.8)	60 (68.2)
Currently married/in union	46 (35.4)	83 (63.9)	68 (88.3)	9 (11.7)	78 (60.0)	52 (40.0)
**Sexually active**					
No	24 (20.9)	88 (76.5)	48 (80.0)	12 (20.0)	53 (46.1)	62 (53.9)
Yes	33 (30.8)	73 (68.2)	48 (87.3)	7 (12.7)	54 (50.5)	53 (49.5)
**Ever given birth**					
No	15 (19.2)	61 (78.2)	24 (80.0)	6 (20.0)	28 (35.9)	50 (64.1)
Yes	42 (29.2)	100 (69.4)	72 (84.7)	13 (15.3)	79 (54.9)	65 (45.1)
**Parity**					
Only 1	10 (18.5)	42 (77.8)	30 (96.8)	1 (3.2)	30 (55.6)	24 (44.4)
2–3	27 (44.3)	34 (55.7)	33 (82.5)	7 (17.5)	39 (63.9)	22 (36.1)
>3	5 (17.2)	24 (82.8)	9 (64.3)	5 (35.7)	10 (34.5)	19 (65.5)
**Media exposure**					
No	7 (9.5)	63 (85.1)	14 (58.3)	10 (41.7)	18 (24.3)	56 (75.7)
Yes	50 (33.8)	98 (66.2)	82 (90.1)	9 (9.9)	89 (60.1)	59 (39.9)
**Woman's decision-making power**					
No power	9 (22.5)	30 (75.0)	16 (72.7)	6 (27.3)	19 (47.5)	21 (52.5)
Have power	48 (26.4)	131 (71.98)	80 (86.0)	13 (13.98)	88 (48.4)	94 (51.6)

### Factors Associated With Exposure to FP Information

From the bivariate analysis, the woman's age, marital status, history of ever giving birth, discussing FP with a field or health worker, and the woman's decision-making power were significantly associated with a higher prevalence of being exposed to FP messages through media (Table 5). After adjusting for statistically significant factors from the bivariate analysis (age, marital status, ever giving birth, receiving FP counseling from a field or health worker, and the woman's decision-making power), only discussing FP with a field or health worker remained significant. The proportion of women who have been exposed to FP messages through media was 1.4 times higher if the woman had discussed FP with a field or health worker (aPR 1.4, 95% CI: 1.24–1.56).

**Table 5:** Factors associated with exposure to family planning messages through media^*^.

**Variable**	**Category**	**Crude PR (CI)**	**Adjusted PR**
Current using FP	No	Ref	
	Yes	1.0 (0.90–1.17)	
Intention to use FP	No	Ref	
	Yes	1.2 (0.87–1.72)	
Discontinued use of FP	No	Ref	
	Yes	0.9 (0.80–1.02)	
Age (years)	15–24	Ref	Ref
	25–34	**1.2 (1.04–1.32)**	1.1 (1.00–1.28)
	35–49	**1.2 (1.01–1.34)**	1.1 (0.99–1.32)
Education level	No education	Ref	
	Primary	1.1 (0.85–1.40)	
	Secondary and higher	1.1 (0.88–1.43)	
Marital status	Formerly/never in union	Ref	Ref
	Currently married/in union	**1.2 (1.05–1.38)**	1.1 (0.93–1.23)
Ever given birth	No	Ref	Ref
	Yes	**1.2 (1.01–1.38)**	0.97 (0.82–1.16)
Parity	Only 1	Ref	
	2–3	0.98 (0.86–1.10)	
	>3	0.9 (0.78–1.05)	
Wantedness of last birth	No child	Ref	
	Later	1.1 (0.89–1.37)	
	Then	1.1 (0.94–1.37)	
Fertility preference	No child/can't get pregnant	Ref	
	Another child/ undecided	1.1 (0.97–1.26)	
Contact with FP provider	No	Ref	Ref
	Yes	**1.4 (1.27–1.59)**	**1.4 (1.24–1.56)**
Woman's decision-making power	No power Have power	Ref **1.3 (1.03–1.63)**	Ref 1.2 (0.95–1.48)

*PR, prevalence ratio; CI, confidence interval*.

* *Includes all four media channels of radio, television, newspaper/magazine, and mobile phone*.

*Bold values are the significant values*.

### Factors Associated With Contact of Non-contracepting Women With FP Providers

Table 6 shows the relationship between overall contact of non-contracepting women with either a field or health worker and sociodemographic factors. The bivariate analysis shows higher prevalence of FP provider counseling among women who were married or in union, women that had ever given birth, and those that had been exposed to media messages. After controlling for the woman's age, education level, marital status, ever giving birth, parity, media exposure, and decision-making power, counseling by an FP provider was associated with marital status and having been exposed to media messages. Prevalence of being counseled by FP providers was 1.6 times higher among women who were married or in union (aPR 1.6, 95% CI: 1.01–2.68) and 2.5 times higher among women who reported receiving FP messages through media (aPR 2.5, 95% CI: 1.37–4.54) compared with those who were not married or in union and those who reported no exposure to media FP messages, respectively.

**Table 6:** Factors associated with FP counseling among non-users of contraception.

**Variable**	**Category**	**Crude PR (CI)**	**Adjusted PR**
Age (years)	15–24	Ref	Ref
	25–34	1.0 (0.73–1.42)	0.8 (0.57–1.14)
	35–49	1.1 (0.80–1.54)	0.99 (0.67–1.49)
Education level	No education	Ref	Ref
	Primary	1.0 (0.55–1.92)	1.0 (0.60–1.71)
	Secondary and higher	1.3 (0.72–2.34)	1.1 (0.69–1.85)
Marital status	Formerly/never in union	Ref	Ref
	Currently married/in union	**1.9 (1.35−2.64)**	**1.6 (1.01–2.68**)
Sexually active	No	Ref	
	Yes	1.1 (0.83–1.44)	
Ever given birth	No	Ref	
	Yes	**1.5 (1.10–2.13)**	
Parity	only 1	Ref	Ref
	2–3	1.2 (0.85–1.56)	1.0 (0.75–1.44)
	>3	0.6 (0.36–1.08)	0.7 (0.37–1.23)
Media exposure	No	Ref	Ref
	Yes	**2.5 (1.62–3.77)**	**2.5 (1.37–4.54)**
Woman's decision-making power	No power Have power	Ref 1.0 (0.71–1.46)	Ref 0.8 (0.59–1.20)

*PR, prevalence ratio; CI, confidence interval*.

*Bold values are the significant values*.

## Discussion

A household survey was conducted to explore knowledge and use of FP among women living in an informal settlement in Uganda. In this study, we assessed the knowledge of the women about FP methods and the prevalence of media exposure to FP messages. We also examined FP counseling for women who were not using contraception at the time of the survey by FP providers. Findings reveal high levels of knowledge of FP methods and where to obtain them but a low prevalence of use and high levels of exposure to media-based FP information but low utilization of a mobile phone channel. In addition, we found low provider-based dissemination of FP information among non-contracepting women, particularly unmarried women and those with no media exposure to FP information.

Increasing knowledge of FP is continuously emphasized in many FP programs as a strategy for increasing uptake or creating demand for FP. Indeed, it has been posited as a key component in effecting behavior change (28, 29). Knowledge about contraception influences perceived benefits or barriers of contraception use, which, in turn, influence contraceptive behaviors of individuals, including method choice (30). The high percentage of all women who knew any FP method and supply source for modern contraceptives found in this study suggests the presence of some cognitive accessibility to FP services in this urban population. This may be explained by the exposure to media messages about FP or discussion of FP by health care workers during client encounters. Also, informal discussions in the women's networks, including discussions with their partners, are potential catalysts to women's knowledge of FP methods. High knowledge of FP methods among users has been widely reported in sub-Saharan Africa (31–33). Our study results also show higher awareness of short-acting compared with long-acting reversible contraceptives. This presents a potential risk for increased occurrence of unintended pregnancies secondary to inconsistent use of short-acting methods (34).

Similar to our finding, studies assessing knowledge and use of contraception have shown a much lower percentage of all women using any contraceptive compared with those who report knowing any FP method (32, 33, 35). However, assessment of knowledge in this study was limited to a woman ever hearing about a specific method, which may not be a complete reflection of the women's understanding of the methods. Contraceptive use is influenced by a wide range of factors, including misconceptions and sociocultural norms (36, 37). The recurrently observed difference between knowledge and use of any contraception calls for further exploration of users' understanding of FP methods and the fit between existing education programs and users' knowledge needs on FP (28). This would involve assessing issues such as content of information disseminated to the women and capacity of women to process, translate, and use the information. Obtaining such information would help design improved programs to support uptake and correct use of FP. Sustainable Development Goal 3.7 aims at ensuring universal access to sexual and reproductive health, including FP. According to the United Nation's population division, the target is to have 75% of existing demand for FP being satisfied by modern methods (18). Tilahun et al. (35) highlight the inadequacy of awareness about contraceptives in meeting FP need and indicate that formal education increases the likelihood of having good knowledge of contraceptives.

Nonetheless, we acknowledge that achieving behavior change requires more than increasing people's knowledge and understanding of FP. Thus, implementation of other complementary strategies such as community group engagement (CGE) and training user groups to impart skills for correct use, as well as creating an enabling environment to support adoption of desirable behaviors, should also be considered (38, 39). With a focus on perceived local drivers and barriers, CGE in dialogue and action is a recognized high-impact practice in FP for behavior change that has the promise to improve women's and men's FP knowledge, improve women's decision-making power, and fostering family or social changes (40). Specifically looking at adolescents and young people within urban spaces, they face various FP challenges and play a wide range of roles, including students, laborers, spouses, or parents (41–43). Besides strengthening the provision of youth-friendly services in marginalized urban areas, the use of benefit cards/vouchers could be used to facilitate equitable access to more contraceptive options through both public and private providers. The vouchers/benefits cards can also be used to enable access to other complementary services, such as pregnancy testing and HIV counseling and testing among others. Some considerations for implementation of benefits cards/vouchers include having clear eligibility criteria, integrating the voucher/cards system into social behavior change strategies, and ensuring equitable distribution. In addition, youth engagement through health and socioeconomic activities may contribute to demand generation and increase uptake of FP in young people (44–46). Additionally, the literature also shows that youths are commonly influenced by peers and other key influential individuals who can be trained and used to disseminate and impart accurate knowledge or inspire positive behavior in adolescents and young people (45, 47). A study by Catwright and colleagues further highlights the use of younger providers and increasing the opening hours for FP clinics, which can motivate this subpopulation to access FP services or methods (47).

Mass media has the potential to influence the contraceptive behaviors of a wide range of individuals by providing a stimulus for considering contraception use (30). About 7 in 10 women in this study had been exposed to FP messages through media, predominantly television and radio. This may explain the high level of awareness observed in the study population. However, the results show no significant association between exposure to media FP messages and contraceptive use or intentions. Although some earlier studies report increased likelihood of using FP with access to media (32, 48, 49), others indicate very weak, inverse, or no relationship between exposure to media and use of FP (50, 51). This study further explores disparities in exposure to FP information through media and finds higher prevalence of exposure among women that had contact with an FP provider. This may be explained by potential support and encouragement given to women during discussions with the providers that motivates them to continue educating themselves through media or general awareness creation by the providers about the different messages in the media. However, in this study population, the proportion of women who had been visited by a field worker with regard to FP was only 22.5%, and the proportion visiting a health facility in the prior year for care for themselves or their children was just over 50%. Also, the bivariate analysis showed lower proportions of exposed women among adolescents and young women, women who had never been married or were not in a union at the time of the survey, women who had never given birth, and those with no decision-making power. These may need to be targeted in demand-creation activities.

The study found a low prevalence of exposure to mobile phone FP messages. We argue that this low proportion is a missed opportunity in meeting the need for FP exposure and linkage to services among these urban residents. People living in informal settlements of urban areas are highly mobile and have inequitable access to FP services (52). Mobile health (mhealth) interventions can help address some of these challenges by providing appropriate information and linkage to care. Studies have explored the potential for mhealth interventions regarding different elements of FP (53–57), and lessons from these can be used to design tailored models for this urban population to boost their capability and provide support for contraception use. The content for these mobile phone–based messages may include information on the benefits and expected side effects of different contraception options, availability of FP methods and services, guidance on reporting and managing common side effects associated with FP use, guidance on initiation or safe switching between methods and sexual and reproductive health rights among others (56–58). The duration and frequency of the messages as well as content should be tailored to the targeted population to improve usefulness or effectiveness, including use of translated voice messages (56). The design may also consider incorporation of motivational messaging and allow for interactive communication (59). Ownership of mobile phones is increasing, at a prevalence of 71% in Uganda (60–62). The adoption of mhealth strategies for FP, however, raises some ethical concerns, such as confidentiality or data security. These can be addressed at different phases using various strategies, such as anonymization, understanding pathways through which risks arise, and effective monitoring for real-time adaptation (63). The low coverage of mobile phone messages may also be due to language barriers, whereby messages are sent in a language that is poorly understood by the users or limited capacity of users to utilize internet-based services.

At least 50% of women in this study were not using any contraception (and were not pregnant), and the results show low reach of this subpopulation by field FP workers. This points to possible missed opportunities in expanding reach of FP services in this urban setting. The usefulness of field FP workers, commonly termed community health workers (CHWs), in supporting FP through education and distribution of commodities has been demonstrated (64–67). The integration of CHWs into the health system is considered a high-impact practice for FP, and this needs to be leveraged in this urban setting (65). CHWs are able to provide contraceptive information and services to underserved or hard-to-reach subpopulations, resulting in improved knowledge and attitudes as well as increased access to and demand for FP services. The results also show lower likelihood of contact with an FP provider among women who were not married/in union and those who had no exposure/access to mass media. This suggests potential bias in the provision of FP information or other services or misperceptions by which not being in a union is perceived as not being at risk of becoming pregnant. A similar finding was reported in Niger, where CHWs were less likely to visit nulliparous women (64). These results have implications for the uptake of FP.

This study contributes to the knowledge base on knowledge about FP among residents of informal settlements in urban areas in Africa. It provides useful insights for design of demand-creation strategies targeting urban populations. Nonetheless, the study is limited by the depth or meaning of knowledge assessed. The assessment of knowledge only asked if the respondent had ever heard about the method, which does not necessarily mean that the person knows what the method actually is or its appropriate use. Also, assessment of media exposure only reported if a respondent had received/read/watched/heard an FP message through media channels within the last few months prior to the survey. This does not include specifics of when the exposure happened, dose of messages delivered/accessed, or in what forms/language the messages are conveyed, which would enable better exploration of the relationship between contraceptive behaviors and media exposure.

## Conclusions and Recommendations

There is high general awareness about FP methods and prevalence of exposure to media-based FP information. Further exploration of women's understanding of FP methods and the fit between existing education programs and FP knowledge needs in this urban setting should be conducted. Tailored education programs should target adolescents and young women, women who do not often visit formal health facilities, nulliparous women, and those living on their own. There is need for innovative solutions to optimize potential of mass media in accelerating progress in reproductive health toward universal access. In addition, health worker contact should be encouraged to enhance the knowledge on contraceptive methods among residents of informal settlements.

## Data Availability Statement

The raw data supporting the conclusions of this article will be made available by the authors, without undue reservation.

## Ethics Statement

Ethical approval was obtained from Makerere University School of Public Health Higher Degrees and Research Ethics Committee (HDREC-684). The study was also registered with the Uganda National Council of Science and Technology (HS382ES). Permission to interview participants was sought from Wakiso District local government and the Kira Municipality officials. Prior to any interviews, informed written consent to participate in the study was sought from all adults above 18years, the legal age in Uganda. For minors (15–17 years), informed consent to participate in the study was first obtained from their parents/guardians, as well as assent from the respondents. Minors who were pregnant or those who had given birth at the time of the interviews were considered emancipated and thus consented on their own.

## Author Contributions

MT conceptualized the study. MT and TS participated in data collection. CB conducted the data analysis and led manuscript development. DC participated in the manuscript development process. MB and JA supported the data analysis process. LA contributed to the conceptualization of the study and reviewing the manuscript. FM provided overall technical guidance to the conceptualization process. MOS provided technical guidance to the manuscript development process. All authors reviewed the manuscript and provided substantial input and approved the final manuscript.

## Conflict of Interest

The authors declare that the research was conducted in the absence of any commercial or financial relationships that could be construed as a potential conflict of interest.
